# Case Report: “Area of Focus” Atypical Trichinellosis and Fascioliasis Coinfection

**DOI:** 10.3389/fmed.2022.881356

**Published:** 2022-05-11

**Authors:** Meng Wang, Wei Liu, Ziman Xiong, Zhen Li, Jiansha Li, Xin Xu, Meng Zhang, Mingyou Xing, Qin Ning, Di Wu, Junying Qi

**Affiliations:** ^1^Department of Nephrology, Tongji Hospital, Tongji Medical College, Huazhong University of Science and Technology, Wuhan, China; ^2^National Medical Center for Major Public Health Events, Department and Institute of Infectious Disease, Tongji Hospital, Tongji Medical College, Huazhong University of Science and Technology, Wuhan, China; ^3^Department of Radiology, Tongji Hospital, Tongji Medical College, Huazhong University of Science and Technology, Wuhan, China; ^4^Department of Pathology, Tongji Hospital, Tongji Medical College, Huazhong University of Science and Technology, Wuhan, China

**Keywords:** case report, *Trichinella spiralis*, *Fasciola hepatica*, magnetic resonance imaging (MRI), metagenomic next-generation sequencing (mNGS)

## Abstract

Parasitic co-infection is commonly observed in natural populations, yet rare in the laboratory. Multiparasitism can have negative effects on the host, ranging from the atypical manifestations to increased mortality, consequently, it may be misdiagnosed and treated with unsuitable anthelmintic medicines. Therefore, reliable diagnosis is critical for appropriate treatment of parasitic co-infection. Herein, we report a case of a 31-year-old woman with persistent eosinophilia and hypoechoic liver lesion on ultrasound. The microscopic examination of multiple stool specimens did not find any pathogens. The patient had serum specific anti-*Trichinella* IgG antibody by Dot enzyme-linked immunosorbent assay (Dot-ELISA). After treatment with albendazole, contrast-enhanced magnetic resonance imaging (MRI) revealed more lesions in the liver. Subsequently, liver biopsy was performed in this patient and *Fasciola hepatica* was identified using metagenomic next-generation sequencing (mNGS) as well as polymerase chain reaction. After treatment with triclabendazole, which is the only anthelmintic drug specifically available against this fluke, her eosinophil count returned normal, and the liver lesions were significantly regressed. This case highlights the diagnostic challenge posed by parasitic co-infection, which merits more in-depth evaluation to confirm the diagnosis.

## Introduction

Albeit holistic implementation of interventions in the control and prevention of parasitic infection, parasitic disease is still prevalent, particularly in the least developed countries, which are attributed to the environmental factors such as animal migration, human factors including poor sanitation, malnourished status, unhealthy social and dietary habits, and the advanced detection methods (1–3). People who have weakened immunity or immune deficiency are susceptible to parasitic infection through consuming contaminated food or water (4). Trichinellosis is a parasitic disease caused by human intake of raw or undercooked meat infected by *Trichinella spiralis* (*T. spiralis*) larvae (5). The China CDC survey reported an increasing occurrence of foodborne parasitic diseases where trichinellosis is ranked as one of the top three (6), posing a great threat to human health. *T. spiralis* primarily parasitizes the striated muscles and small intestine, causing a broad spectrum of clinical symptoms and signs such as eosinophilia, fever, abdominal pain, myalgia etc. It may also affect hepatic parenchymal and lead to localized lesions. Recently there have been some case reports of a typical radiological feature in hepatic trichinellosis in liver magnetic resonance imaging (MRI), manifesting as “curved tunnel” sign. Such patients may be initially misdiagnosed with hepatic tumor. Some cases of trichinellosis were confirmed by histopathological examination after hepatectomy surgery. The early identification of characteristic manifestations in liver MRI may avoid unnecessary invasive operations (7).

Microscopy remains the mainstay of laboratory diagnostic testing for parasitic infection (8), but its sensitivity largely relies on the number of parasites and their distribution in the sample, the sampling procedures, and the technical skills of laboratory professionals. The co-infection of other parasites, either conspecific or heterospecific, is often neglected and makes the clinical diagnosis more difficult. The patients who have parasitic co-infection commonly present with atypical clinical symptoms and elevated levels of non-specific serological markers such as eosinophil, allergy-related parameters, inflammatory markers etc (2, 9, 10). In the meantime, the interactions between the parasites and the host might alter the evolution of parasite virulence and the host immunity, thereby potentially leading to diagnostic and therapeutic delays (11, 12). Therefore, early recognition of co-infection is of critical importance for timely treatment. In clinical practice, if empiric and diagnostic-driven anthelmintic treatment does not improve patient's outcome, probable parasitic co-infection should be taken into consideration, and additional diagnostic tests or procedures will be necessary. Currently, metagenomic next-generation sequencing (mNGS) technology has proven capabilities of detecting a wide range of pathogens, and has emerged as a promising unbiased culture-independent pathogen detection technique for infectious disease diagnostics (13, 14). In this study, mNGS was used to confirm fascioliasis coinfection in a patient who has been previously diagnosed with atypical trichinellosis.

## Case Description

**Figure 1:**
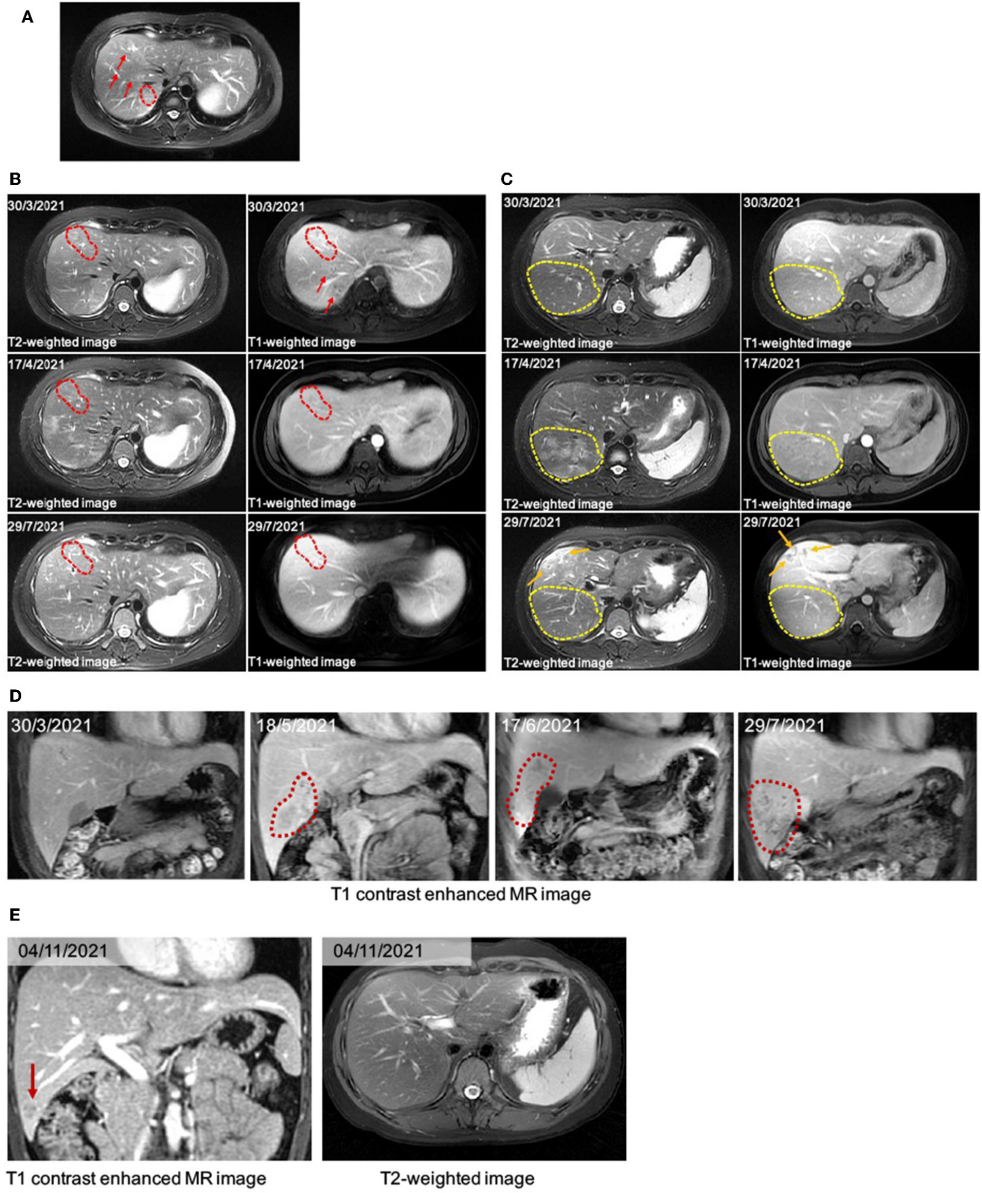
**(A)** The liver MRI image. There are several typical curved tunnel signs (red arrows and dotted frame) on T2WI. **(B)** A serial image of the patient before and after anti-helminthic therapy in the hepatic anterior lobe. The image on March 30^th^, 2021, showed typical “curved tunnel” signs in hepatic right anterior and posterior lobe on T1WI and T2WI image (red dotted frames and arrows). On April 17^th^, a month after the first therapy, the area of curved tunnel signs in hepatic anterior lobe decreased. After the following anti-helminthic treatments, the liver MRI image showed further decreased signs (29/7/2021). **(C)** A serial image showed dynastic change in the hepatic posterior lobe. On the image of March 30th, there were several curved tunnels in the marked yellow dotted frame. The new massive patchy slight hyperintense signals on April 17^th^ were almost gone on the image of July 29^th^. Besides, we observed newly tunnel changes located under the subcapsular (orange arrows). **(D)** Coronal image showed subcapsular lesion appeared first on the image of May 18^th^, 2021. During the anti-helminthic therapy course, the lesion expanded and was progressed into abscess. **(E)** On the T1 contrast enhanced MR image, the subcapsular lesion in hepatic right lobe decreased significantly (red arrows).

A 31-year-old female office worker presented with persistent eosinophilia. The absolute eosinophil count was 0.34 × 10^9^/L and percentage of eosinophil was 6.3% when she did routine screening tests in pregnancy on December 28, 2020. The patient was otherwise healthy and was not on any medications. She was asymptomatic and denied any fever, rash, abdominal pain and myalgia, as well as any animal exposures, drug use, travel history, or family history of inflammatory diseases. But she used to eat scalded or grilled pork which might be undercooked. The repeat smear microscopic examinations revealed no parasites, ova (eggs) or cysts in her fecal samples. Then she was screened for parasitic infection using an in-house IgG Dot enzyme-linked immunosorbent assay (Dot-ELISA, Jackson Immuno, Lot number: 104541; detailed method was listed in Supplementary Material) and tested positive for anti-*T. spiralis* IgG. Because of pregnancy, she did not receive any anthelmintic treatment but regular hospital visits. After delivery, she was admitted to the hospital for extensive diagnostic evaluation and intervention. On March 9, 2021, her eosinophil count and percentage reached 11.37 × 10^9^/L and 72.0%, respectively. Bone marrow aspiration revealed increased proportions of eosinophils (35%), without clonal proliferation of eosinophil precursors. However, the FIP1L1-PAGFRα gene test, a clonal marker for hypereosinophilic syndrome (HES) or chronic eosinophilic leukemia (CEL), was negative. The routine abdominal color doppler ultrasonography (CDU) revealed a 4.1 × 2.0 cm hypoechoic area in posterior right hepatic lobe, suggesting possible focal hepatosteatosis. Subsequently, liver MRI with routine T1- and T2-weighted images (axial and coronal panel) and diffusion-weighted images (DWI) was performed showing typical curved tunnel sign in right hepatic lobe (Figure 1A), consistent with the abnormal lesion on CDU image. Serological tests showed that alamine aminotransferase (ALT), aspartate transaminase (AST), albumin and globulin were within normal range. The levels of inflammation markers including c-reactive protein (CRP), erythrocyte sedimentation rate (ESR) was mildly elevated. Serum tumor marker levels were within the normal range. MRI of the brain and bilateral calves did not show any abnormal signs. Based on these results, the patient was initially diagnosed with hepatic trichinellosis. On March 20, 2021, she received 5-day course of treatment with albendazole (600 mg twice daily), and concomitant prednisolone (10 mg once daily) to blunt inflammatory response to dying parasites. Thereafter during the next 4 months the patient received long-term follow-up and intermittent administration of albendazole or praziquantel on a regular basis (almost every month).

After anthelmintic treatment with albendazole or praziquantel, there was no significant decline in her absolute eosinophil count. Serological markers remained within the normal range. However, the immunoglobulin E (IgE) kept increasing, finally exceeding the upper detection limit of 2,500 IU/mL. The liver MRI images showed decreased tunnel signs in both anterior and posterior segments of the right hepatic lobe. The T2-weighted image (T2WI) showed new patchy hyperintense lesion in right hepatic lobe after the first anthelmintic treatment, which was indictive of inflammatory effusion and edema caused by larvae of parasites. The MRI results were interpreted by two senior consultant radiologists who had no knowledge of the initial ultrasound reports. On July 29, 2021, the MRI revealed that the above lesion had shrunk significantly (Figures 1B,C), suggesting that the hepatic injury possibly induced by *T. spiralis* might be improved after effective anthelmintic treatment.

**Figure 2:**
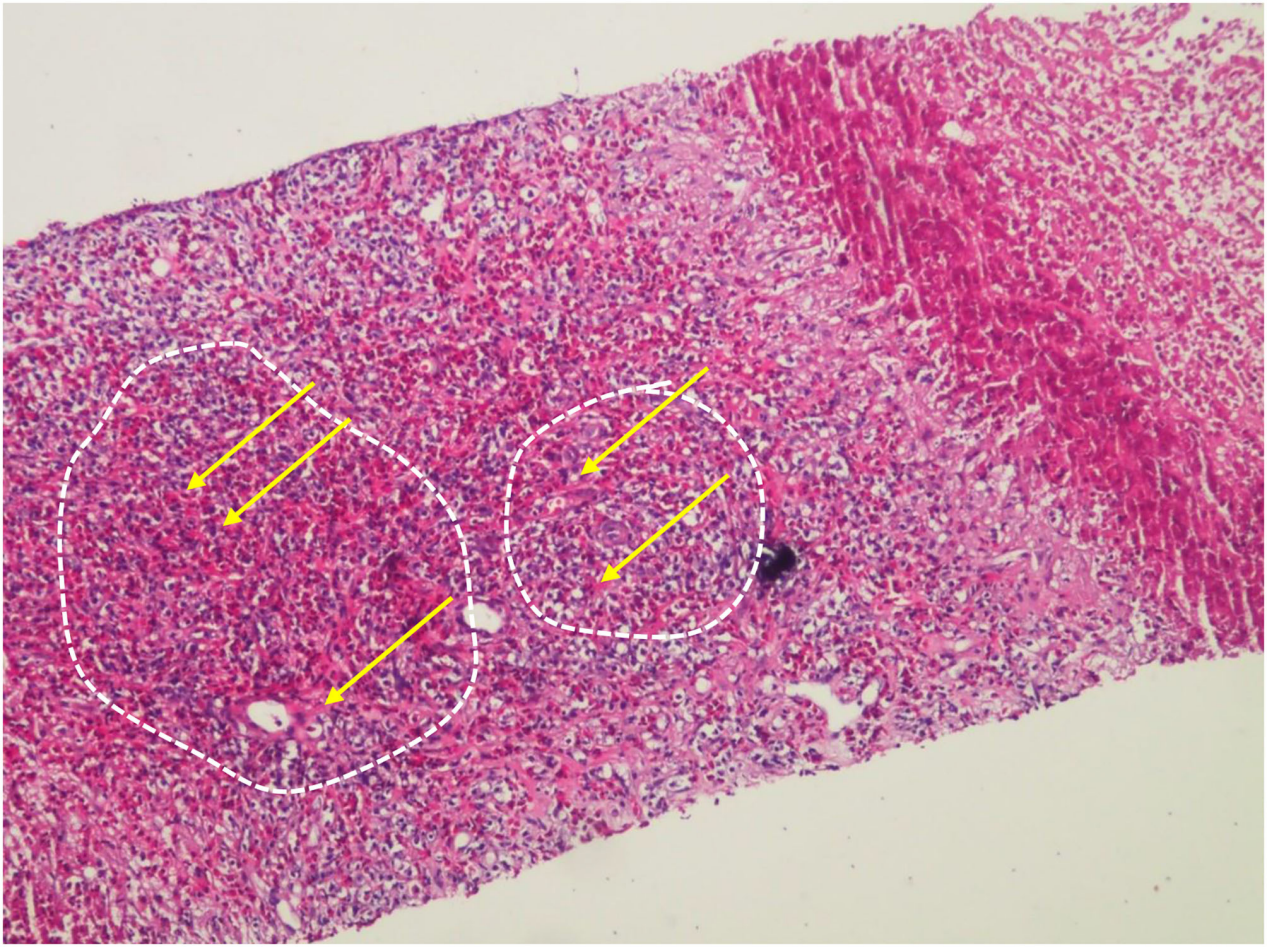
HE staining (400X) showed massive eosinophilic infiltration and abscess formation. The dashed white box indicates abscesses and the yellow arrow indicates eosinophilic infiltration.

**Figure 3:**
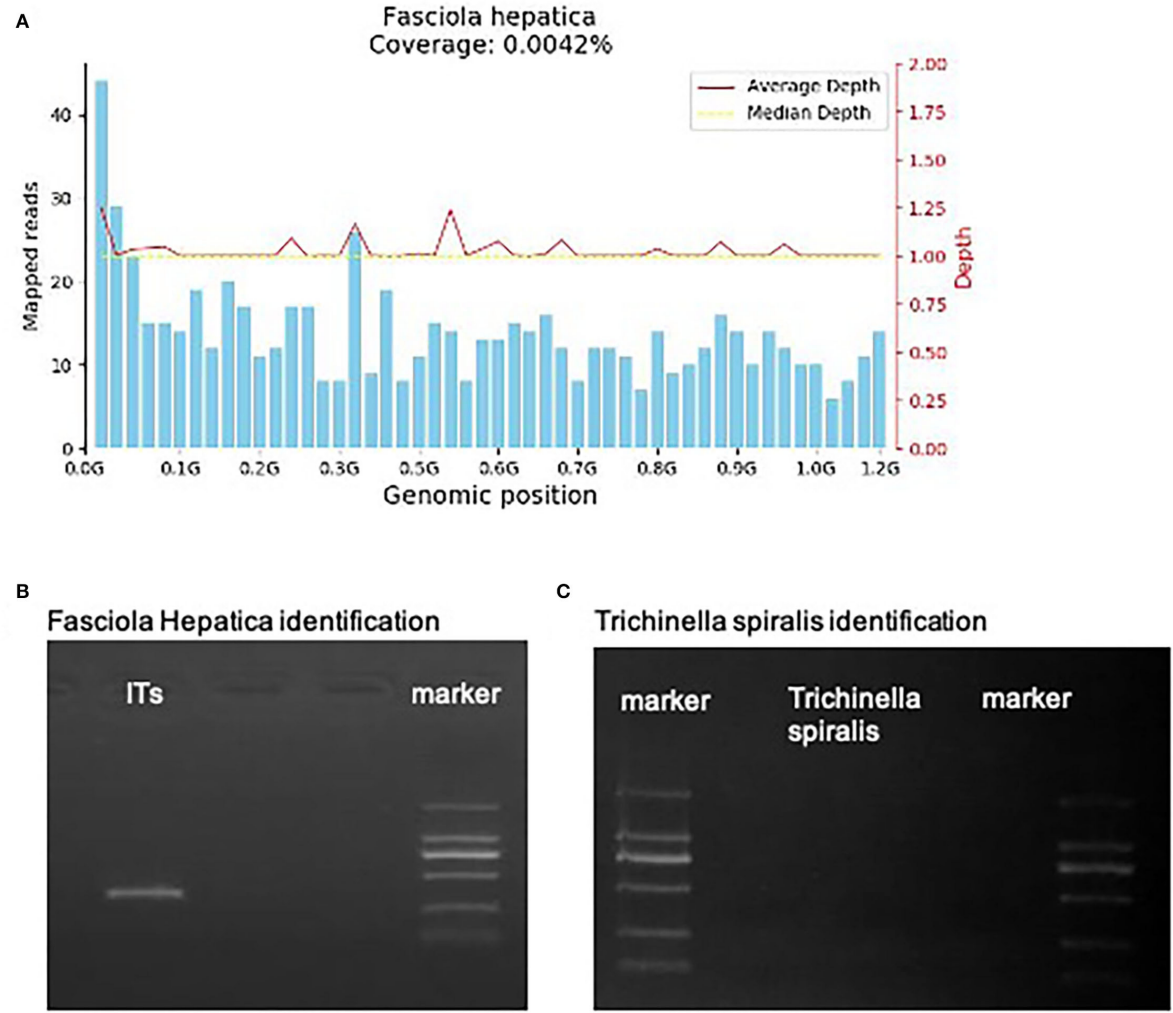
**(A)** The mNGS result. 1,299 specific *Fasciola hepatica* sequences that covered 0.0042% of the total *Fasciola hepatica* genome were detected by mNGS in the hepatic sample of the patient. The sequences were symmetrical matched with the median depth. **(B,C)** *Fasciola Hepatica* and *Trichinella spiralis* identification of gene amplification.

**Figure 4:**
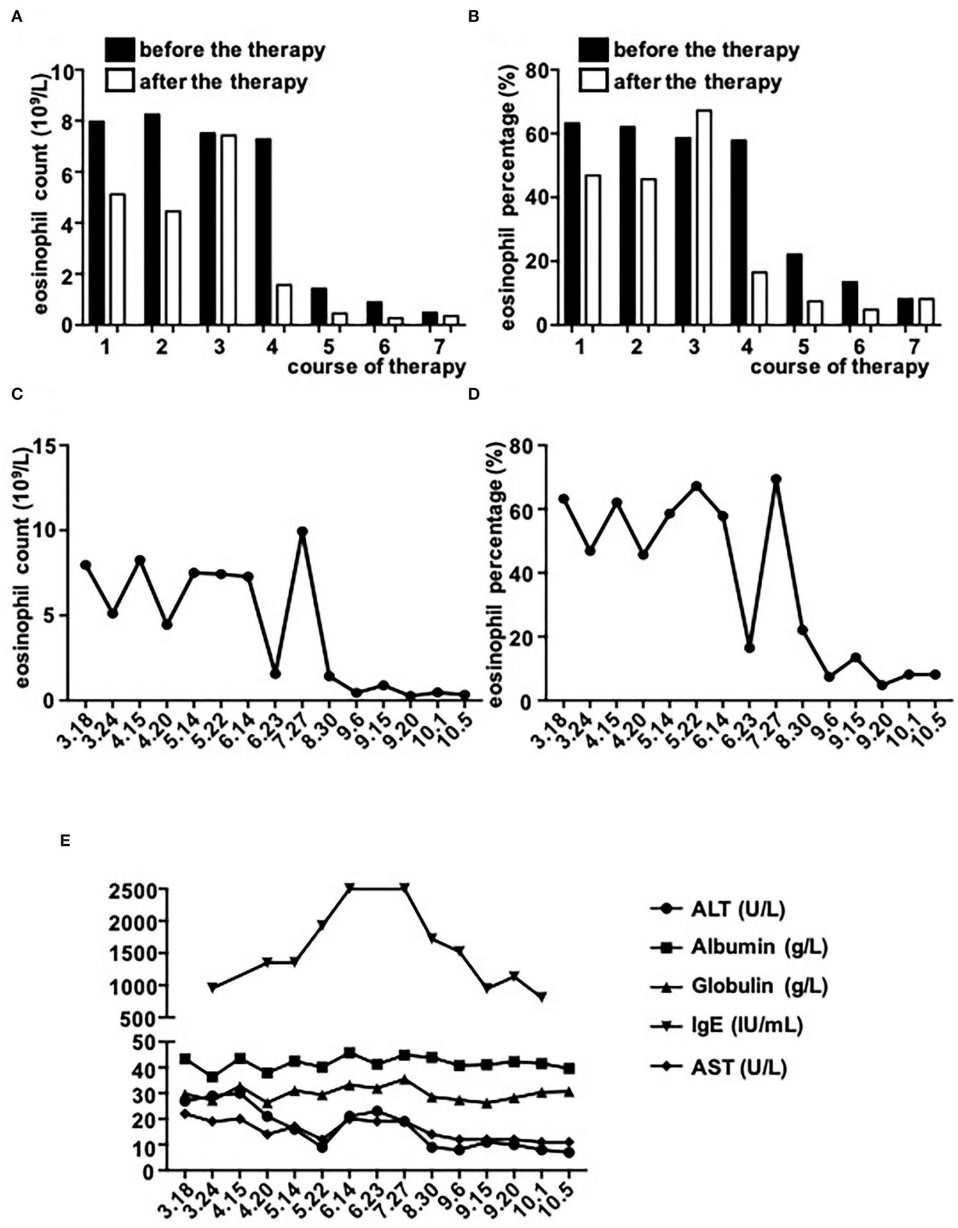
The dynamic changes of serological markers. **(A–D)** Eosinophil counts and percentages before and after every therapy (each therapy course: 3.18–3.24/4.15-4.20/5.14–5.22/6.14–6.23/8.30–9.6/9.15–9.20/10.1–10.5). The number of Eosinophil decreased after four therapies (June 23^th^). The count of Eosinophil showed a transient and significant increase on the measurement of July 29^th^, then kept within low level. **(E)** The trend of other markers. The level of IgE kept increasing and tripled compared with the first measurement. After four therapies, the level of IgE gradually went down. ALT, AST, albumin, and globulin kept within normal range.

However, on May 18, 2021, the coronal T1-weighted image (T1WI) showed newly-emerged hypointense lesion in anterior segment of the right hepatic lobe, which was localized in close proximity to the portal vein and bile duct system. After intermittent treatment, on July 29, 2021, the lesion extended that had a similar appearance to the liver abscess (Figure 1D). Therefore, this patient underwent an ultrasound-guided percutaneous liver biopsy to clarify the diagnosis. The histopathological examination showed massive eosinophilic infiltration generating abscess or granuloma (Figure 2). Liver sample was also collected to identify the pathogen using metagenomic next generation sequencing (mNGS) technology, the number of species-specific reads aligning to the *Fasciola hepatica (F. hepatica)* genome in the liver biopsy was 1,299 (Supplementary Table 1, Figure 3A), which was further confirmed by polymerase chain reaction (PCR) (Supplementary Table 2, Figures 3B,C). We collected detailed medical history and the patient had a habit of eating raw vegetables. The final diagnosis of this patient was trichinellosis and fascioliasis coinfection. The patient was treated with two single doses of triclabendazole 10 mg/kg on October 2, which is the first-line therapy against fascioliasis. The eosinophil count returned to normal 1 month after triclabendazole treatment (Figure 4), and the liver MRI showed that hepatic lesion was significantly regressed in anterior segment of the right hepatic lobe (Figure 1E), suggesting that the treatment was effective.

## Discussion

To our knowledge, this is the first case report of trichinellosis and fascioliasis coinfection. Fascioliasis is a zoonotic parasitic disease caused by two major trematodes, *F. hepatica* and *Fasciola gigantica*. Human infection is typically acquired by ingesting metacercaria contaminated water or aquatic plants. *F. hepatica* has temperate and tropical distribution. In China, the laboratory-confirmed cases are mainly scattered in Guangxi, Yunnan, Guizhou, and Qinghai Province (14). Until now, *F. hepatica* infection has rarely been reported in Wuhan. However, the movements of livestock allow the infection spreading from endemic to non-endemic areas. Unlike fascioliasis, trichinellosis is caused by the consumption of raw or undercooked meat containing *T. spiralis* larvae. Although previous studies demonstrated that *T. spiralis* larvae do not invade the liver, currently there were some case reports of trichinellosis with liver involvement (7, 15). The mechanisms underlying trichinellosis associated hepatic injury remains unclear, probably by physical injury and inflammation when larvae pass through the liver (16, 17). Moreover, *T. spiralis* co-infection may exacerbate hepatopathy induced by other parasites (18). On the contrary, *F. hepatica* can invade liver directly. The newly excysted juvenile flukes migrate through the intestinal wall to the peritoneal cavity, and then penetrate the Glisson capsule to enter the liver and eventually the bile ducts where they mature, leading to inflammation, abscess formation, hemorrhage, necrosis, granulation, fibrosis, and biliary obstruction (19). Patient may present with fever, jaundice, biliary colic, right hypochondriac pain with gastrointestinal symptoms, and a small proportion of patients are asymptomatic, which is different from trichinellosis with respect to clinical manifestations (20).

The serodiagnosis of human trichinellosis or fascioliasis is mainly established by detecting antibodies using ELISA or indirect fluorescence antibody test (IFAT). In some cases, the definitive diagnosis of trichinellosis can be made by detecting larvae in a biopsy muscle samples, but the technique is invasive and unable to detect the early stage of infection. However, clinical symptoms and laboratory findings such as eosinophilia, increased total IgE and muscle enzyme levels may help in diagnosis (5). Unlike trichinellosis, microscopic examination of stool samples or duodenal bile aspirates for ova can be used for the clinical diagnosis of fascioliasis, but is often unrevealing during the acute phase of infection (21). Besides, invasive techniques including endoscopic retrograde cholangiopancreatography (ERCP), percutaneous cholangiography, and liver biopsy, may aid in the diagnosis but are not essential (22). Radiological examinations, including CDU, computerized tomography (CT), as well as MRI are used widely for confirmation and follow-up of fascioliasis.

There are some similarities and differences between MRI findings of fascioliasis and trichinellosis with liver involvements. Both can reveal multiple tunnel-like hypointense lesions on T1WI and hyperintense signs on T2WI in the right hepatic lobe which may be caused by parasites movement. However, the lesions observed in MRI induced by *T. spiralis* may have bead-like appearance while in fascioliasis the lesions are more likely to have irregular shapes and appear in clusters. It might be partly attributed to the different shapes of these two parasites (6, 23–25). In some cases of biliary fascioliasis, radiological examination may show intrahepatic or extrahepatic bile duct dilatation, and adult flukes may be detected with ERCP. Nevertheless, the positive detection rate of trichinellosis in the liver sample is low, as adult *T. spiralis* resides in the intestinal tract and the larvae can be found encapsulated in striated muscle cells, whereas they cannot develop in the liver (17, 26).

In addition to the traditional diagnostic tests including smear microscopic examination, serological diagnostic tests and imaging techniques, mNGS was used in this case to confirm the diagnosis. This methodology allows for unbiased identification and genomic analysis of the entire microbial community in the sample. For pathogens that are unculturable or that require lengthy culture periods, mNGS offers several advantages over traditional pathogen detection methods. The application of mNGS for detecting parasitic infection has been reported recently, highlighting the potential of mNGS to identify parasites and evaluate therapeutic effects of anthelmintic treatment (13, 14). Despite recent successes of mNGS, at present mNGS still has some limitations, such as human background, differentiation between infection and colonization, method standardization, and data storage, protection, analysis, and interpretation, etc. In the present study, hepatic lesion in right hepatic lobe was attenuated after albendazole treatment, but newly-emerged lesion in the anterior segment of the right hepatic lobe was significantly regressed after triclabendazole treatment, suggesting that liver involvement in this case is likely caused by fascioliasis. Albendazole and mebendazole are the principal anthelmintic drugs for the treatment of trichinellosis, possessing substantial anthelmintic activity against *T. spiralis* infection both at the enteral and encapsulated phases (27). However, these anthelmintics have relatively low anthelmintic capacity against *F. hepatica* larvae. Consistently, we observed that in the present case, liver lesions extended after anthelmintic treatment with albendazole and praziquantel. Thus, triclabendazole, which is the only chemical that kills early immature and adult *F. hepatica* (28), was administered to this patient. As currently Triclabendazole has not been available in Chinese mainland, the patient purchased Triclabendazole from Hongkong in accordance with the guidance for personal importation of drug. The adjusted treatment demonstrated to be effective.

There are several limitations of this study. First, the patient presented with persistent eosinophilia as initial manifestation, in the absence of abdominal pain or other typical symptoms. At disease onset she was pregnant and reported a history of frequent consumption of undercooked meat, suggesting the possible diagnosis of parasitic infection. However, in the beginning we did not consider the diagnosis of fascioliasis, due to the unavailability of the ELISA test kit for detection of anti-*F. hepatica* antibody as well as negative results of smear microscopic examination of the stool samples. Second, given triclabendazole is effective in treating both adults and larvae of *T. spiralis* and early immature and adult *F. hepatica*, we cannot conclude that the hepatic lesion was mainly caused by *F. hepatica* infection or by both parasites in this case. In addition, it remains unclear whether the coinfection of *F. hepatica* and *T. spiralis* occurred concomitantly or sequentially. Last, although in this case histopathological examination showed massive eosinophil infiltration and remarkable necrosis of the hepatic parenchyma cells, which are typical histologic features related to parasitic infection, *T. spiralis* or *F. Fasciola* larvae were not found in liver biopsy specimen. We suggested that a fecal egg count (FEC) using sedimentation method should be utilized to look for the eggs, which requires a large amount of fecal sample. However, the patient refused to do further stool examination, as well as ERCP procedure, which might provide clear diagnostic clues.

## Conclusion

In clinical practice, parasitic co-infection is not rare and poses a diagnostic challenge because clinical manifestations may be not typically pathognomonic for specific parasites, thus it merits more in-depth evaluation to confirm the diagnosis. mNGS technology offers distinct advantages in detecting co-infection pathogens. Early recognition of co-infection is of critical importance for timely treatment.

## Author Contributions

JQ, ZL, QN, and MX designed the study. Data collection was carried out by MW, DW, WL, JL, ZX, XX, and MZ. The article was written by MW and revised by DW and JQ. The final manuscript was read and approved by all the authors.

## Funding

This work was supported by the National Key Research and Development Program of China (2021YFC2600200).

## Conflict of Interest

Vision Medicals Co., Ltd performed the mNGS. The authors declare that the research was conducted in the absence of any commercial or financial relationships that could be construed as a potential conflict of interest.

## Publisher's Note

All claims expressed in this article are solely those of the authors and do not necessarily represent those of their affiliated organizations, or those of the publisher, the editors and the reviewers. Any product that may be evaluated in this article, or claim that may be made by its manufacturer, is not guaranteed or endorsed by the publisher.
