# Supplementary material for: Case Report: “Area of Focus” Atypical Trichinellosis and Fascioliasis Coinfection
Source: Front Med (Lausanne). 2022 May 11;9:881356. doi: 10.3389/fmed.2022.881356 (PMC9132012; doi:10.3389/fmed.2022.881356)
Supplement: Supplementary file 1 [file Data_Sheet_1.docx]

**Supplementary Table 1. mNGS results of the patient**

| **Specimen** | **Liver tissue** |
| --- | --- |
| Detected pathogens and specific reads | *Fasciola hepatica (1299)*  *Enterobacter cloacae complex (18)* |

**Supplementary Table 2. Primer sequences for PCR**

|  | **Sequences** |
| --- | --- |
| ITsF | GTACCGTCGCTATATGAAAATAGC |
| ITsR | GACATCTTGAACGCATATTGC |
| Trichinella spiralis-F | TTGTAAAGCGGTGGTGCGTA |
| Trichinella spiralis-R | CATAGAGAGGCAACATTACCT |

**The detailed method of Dot-ELISA**

Dot-ELISA was carried out based on the standardized protocols. Generally, nitrocellulose (NC) with 0.22 μm pores were cut into strips of 0.8 mm wide and 5 cm long. Then, Trichinella antigens were dotted on NCs followed by incubation for 45 mins at room temperature. After drying, non-specific protein binding sites were blocked by addition of PBS-M 5% (pH 7.4) before the incubation with serum samples. Then, serum samples were diluted with PBS-M 3% and added to strips. After incubation at 37°C for 45 mins, strips were washed 3 times in PBS-T 0.1%. Bound human IgG was detected by adding goat anti-human IgG horseradish peroxidase (HRP) -labeled conjugates (Jackson Immuno, Lot number: 104541) to the strips. Following incubation at 37°C for 45 mins, strips were washed 3 further times and soaked with a fresh solution of diaminobenzidine substrate (DAB) and incubated for 10 mins, rinsed with distilled water and blotted dry. Each in-house Dot-ELISA strip was tested using serum from each category. Samples with brown spots (compared to negative and positive control sera, antigens, and secondary antibody controls) were reported as positive.
